# Epigenetically silenced miR-34b/c as a novel faecal-based screening marker for colorectal cancer

**DOI:** 10.1038/bjc.2011.82

**Published:** 2011-05-24

**Authors:** M Kalimutho, S Di Cecilia, G Del Vecchio Blanco, F Roviello, P Sileri, M Cretella, A Formosa, G Corso, D Marrelli, F Pallone, G Federici, S Bernardini

**Affiliations:** 1 Department of Internal Medicine, University of Rome ‘Tor Vergata’, Rome, Italy; 2 Department of Laboratory Medicine, UOC Clinical Molecular Biology and Biochemistry, University Hospital Tor Vergata, Viale Oxford 81, Rome 00133, Italy; 3 Department of Internal Medicine, Gastroenterology Unit, University Hospital Tor Vergata, Rome, Italy; 4 Department of Human Pathology and Oncology, Section of Advanced Surgical Oncology, University of Siena, Siena, Italy; 5 Istituto Toscano Tumori, Firenze, Italy; 6 Department of Surgical Oncology, University Hospital Tor Vergata, Rome, Italy

**Keywords:** miR-34b/c, miR-148a, methylation, faeces, CRC

## Abstract

**Background:**

MicroRNAs are tiny non-coding small endogenous RNAs that regulate gene expression by translational repression, mRNA cleavage and mRNA inhibition. The aim of this study was to investigate the hypermethylation of miR-34b/c and miR-148a in colorectal cancer, and correlate this data to clinicopathological features. We also aimed to evaluate the hypermethylation of miR-34b/c in faeces specimens as a novel non-invasive faecal-DNA-based screening marker.

**Methods:**

The 5-aza-2′-deoxycytidine treatment and methylation-specific PCR were carried out to detect the hypermethylation of miR-34b/c and miR-148a.

**Results:**

The miR-34b/c hypermethylation was found in 97.5% (79 out of 82) of primary colorectal tumours, *P*=0.0110. In 75% (21 out of 28) of faecal specimens we found a hypermethylation of miR-34b/c while only in 16% (2 out of 12) of high-grade dysplasia. In addition, miR-148a was found to be hypermethylated in 65% (51 out of 78) of colorectal tumour tissues with no significant correlation to clinicopathological features. However, a trend with female gender and advanced age was found, *P*=0.083. We also observed a trend to lower survival rate in patients with miR-148a hypermethylation with 10-year survival probability: 48 *vs* 65%, *P*=0.561.

**Conclusions:**

These findings show that aberrant hypermethylation of miR-34b/c could be an ideal class of early screening marker, whereas miR-148a could serve as a disease progression follow-up marker.

MicroRNAs (miRNAs) are a class of small regulatory non-coding RNAs of ∼22 nucleotides in length that modulate specific cellular activity post transcriptionally. The miRNAs target the post-translation level of mRNA through a sequence-specific complimentary at the 3′ UTR region of a gene, thus inhibiting gene expression (Lee *et al*, 1993; Wienholds *et al*, 2005). The human genome is encoded by at least 3% miRNAs, and up to 30% of human protein encoding genes may be regulated by miRNA modulation (Sassen *et al*, 2008). The miRNAs are shown to have key roles in normal development and also in differentiation, cell proliferation and apoptosis of cancer cells (Bartel, 2004; He *et al*, 2005). In the miRBase, thousands of miRNA have been bioinformatically predicted for both prokaryote and eukaryote, and awaiting experimental validation. Each of these miRNA could potentially regulate up to several hundred genes at mRNA post-translation level (Krek *et al*, 2005).

Previous study has shown that more than 50% of the miRNA genes are located in cancer-associated genomic regions or in fragile sites which are hot spots for gene deletion, amplification and mutations (Calin *et al*, 2004), suggesting major functions for miRNAs in cancer cell biology. Furthermore, miRNAs also have been shown to be involved in a wide variety of human cancers, including colon, pancreatic, breast, prostate, liver and ovarian cancer, suggesting a more negative regulation of cancer cell growth by miRNAs modulation (Michael *et al*, 2003; Gramantieri *et al*, 2007; Hurteau *et al*, 2007; Lee *et al*, 2007; Shi *et al*, 2007; Barbarotto *et al*, 2008; Felicetti *et al*, 2008; Mitomo *et al*, 2008). Initially, miR-15 and miR-16 were shown to be involved in the pathogenesis of chronic lymphocytic leukaemia (Calin *et al*, 2002), and later He *et al* (2005) and Johnson *et al* (2005) described a relationship between a miRNA cluster of mir-17–92 with Myc oncogenic pathway.

One of the processes that tightly link miRNAs and cancer is the process called epigenetic modification. This epigenetic alteration in cancer has been shown to occur together with the genetic alterations in colorectal cancer (CRC). Thus, the aberrant hypermethylation process drives the initiation and the progression of colorectal polyps towards invasive of advanced stage colorectal tumours. Two recent studies have addressed the involvement of epigenetically silenced miRNA genes in CRC tumourigenesis including miR-34b/c and miR-342. Moreover, miR-148a has also recently been discovered to be involved in metastasis progression in several cancers including in CRC. Therefore, the understanding of the early process of miRNAs methylation signature in CRC is of utmost important to define the actual tumourigenesis process involved, other than that of hypermethylation protein coding tumour suppressor genes (Grady *et al*, 2008; Toyota *et al*, 2008).

Analysing the promoter methylation of cancer-related genes has been always difficult, as it involved multiple steps for the conversion of unmethylated cytosine residues to uracil, but leaves 5-methylcytosine residues unaffected, particularly when analysing the samples derived from tumour specimens and bodily fluids. In this paper, we have applied the whole bisulfitome amplification method based on isothermal multiple displacement amplification technology. A uniquely processive DNA polymerase with a 3′–5′ exonuclease proofreading activity maintains high fidelity during the replication process. This technique was developed using REPLI-g technology to allow reproducible and representative amplification of bisulfite converted DNA, while maintaining the converted sequence representation (for further details can access from http://www.qiagen.com/).

In this study, we investigate miR-34b/c and miR-148a hypermethylation pattern in CRC tissues and correlate it with clinicopathological features. Furthermore, we wanted to confirm whether miR-34b/c could be used as a possible screening marker for the detection of malignant colonocytes in faeces as a novel non-invasive method. We also aimed to determine the suitability of miR-148a as a prognosis factor for CRC. We showed that miR-34b/c could be an ideal candidate target for CRC screening in faecal microenvironment, whereas the involvement of epigenetic silencing factor in miR-148a correlates to poor prognosis.

## Materials and methods

### CRC cell lines and drug treatment

The CRC cell lines used in this study were HCT116, HT29, LoVo and HCT15. All cell lines were tested for mycoplasma infection and were grown in monolayer cultures in DMEM (HCT116, HT29), RPMI 1640 (HCT15) and Ham's Nutrients mix F12 (LoVo) supplemented with 10–20% FBS and 1% penicillin streptomycin according to UKCCCR guidelines (Masters *et al*, 2000) and as previously reported (Kalimutho *et al*, 2010a). No antibiotics were added to the medium before the treatment. The cells were trypsinised and passed twice a week and the experiments were conducted at early passages. Later the cells were treated with 5-aza-2′-deoxycytidine (AZA; Sigma-Aldrich, St Louis, MO, USA) for 5 days.

### Quantitative PCR (qPCR)

#### RNA isolation, cDNA synthesis and RT–qPCR

Total RNA was isolated using miRNeasy kit according to the manufacturer's guideline (Qiagen, GmbH, Hilden, Germany). The integrity of total RNA was determined by 1% agarose gel electrophoresis. Applied Biosystems RT–qPCR primer sets for miRNA-specific reverse transcription (Ambion Inc., TX, USA) including RNU19 and RNU6B was used according to the manufacturer's protocol. Briefly, the reaction master mix containing 5 × RT Buffer, 5 × RT Primer, Array-Script Enzyme Mix (Applied Biosystems, Foster City, CA, USA) and nuclease-free water was mixed with 50 ng of input miRNA. The mixture was incubated for 30 min at 37°C and then for 10 min at 95°C. The qPCR was carried out using the Stratagene Real-Time PCR System (Stratagene, Agilent Technologies, Santa Clara, CA, USA) with Applied Biosystems qRT–PCR miRNA Detection Kit (Ambion Inc.). The PCR master mix containing Applied Biosystems 2 × PCR Buffer (with FAM labelled Taqman probe), 1 *μ*l FAM-labeled-specific primers and cDNA from process miRNA (Applied Biosystems). The RT products was processed as follows: 95°C for 10 min, and then for 95°C for 15 s and 60°C for 30 s for up to 40 cycles (*n*=2). Fold changed expression following AZA treatment was calculated via a 2^−ΔΔ*C*_q_ method.

### Tissues and faecal DNA

The CRC with matched normal tissue samples were obtained from Surgery Unit of Tor Vergata University Hospital and from University of Siena, which were approved by both internal review boards. A total of 81 primary CRC tissues together with 42 matched control fresh frozen tissues were used for DNA extraction. Faecal DNA was extracted from 28 CRC patients, 39 healthy individuals and 12 patients with high-grade dysplasia (HGD) of colorectum.

### Isolation of miRNA and DNA from CRC and normal tissue samples

Approximately 10–15 mg of fresh frozen tissues of CRC and normal surrounding tissues were used to extract DNA using DNAeasy kit following the manufacturer's guideline (Qiagen).

### Bisulfite conversion of genomic DNA and methylation-specific PCR (MSP)

Bisulfite conversion of genomic DNA was performed as described in Epitech Bisulphite Conversion kit (Qiagen) to create a template for MSP. Briefly, 1.5 *μ*g of genomic DNA from each sample in a volume of 20 *μ*l was mixed with an appropriate chemical solvent provided then processed accordingly. Finally, the converted DNA was re-suspended in 40 *μ*l distiled water to a final concentration of 25–30 ng *μ*l^−1^. Bisulfite-treated DNA was then used as template for MSP, which was performed following the methylation-specific primer (Table 1). Briefly, 2 *μ*l of bisulfite-converted genomic DNA served as the PCR template. The 1 × PCR buffer supplemented with 1.5 mM MgCl_2_, 0.25 mM of each primer, 0.2 *μ*M of dNTPs, 0.1 *μ*g *μ*l^−1^ BSA and 0.25 U of Hot Start Taq polymerase (Applied Biosystems) in a total volume of 20 *μ*l. Cycling conditions for methylated and unmethylated strand of miR-34b/c were as follows: preheating at 95°C for 10 min followed by 40 cycles of denaturation at 95°C for 30 s, annealing at 59°C for 30 s and extension at 72°C for 30 s, and a final extension at 72°C for 7 min. For unmethylated strand of miR-148a: preheating at 95°C for 10 min followed by 40 cycles of denaturation at 95°C for 30 s, annealing at 54°C for 30 s and extension at 68°C for 30 s, and a final extension at 68°C for 7 min. For methylated strand of miR-148a: preheating at 95°C for 10 min followed by 40 cycles of denaturation at 95°C for 30 s, annealing at 56°C for 30 s and extension at 72°C for 30 s, and a final extension at 72°C for 7 min. Qiagen's methylated and unmethylated control DNAs served as a reaction control for PCR.

### Preparation of faecal DNA

Faecal DNA was obtained as previously described (Kalimutho *et al*, 2010b). Faecal DNA of average-risk individuals with no previous history of colon cancer or polyps (*n*=39), CRC patients (*n*=28) and polyps with HGD (*n*=12), all provided written informed consent. DNA from each sample was then subjected to bisulfite conversion and then whole bisulfitome modification later for MSP. All assays were carried out in a blind manner by two different technicians.

### Whole bisulfitome amplification of bisulfite-converted faecal DNA

Faecal DNA whole bisulfitome amplification was carried out using Whole Bisulfitome kit (Qiagen). This assay is based on REPLI-g technology to allow reproducible and representative amplification of bisulfite converted genomic (smaller DNA fragment size and changed nucleotide composition because of the bisulfite conversion), while maintaining the converted sequence representation. Briefly, bisulphited-converted DNA was added with REPLI-g Midi DNA Polymerase (Qiagen) and incubated at 28°C for 8 h. Later the reaction mixtures were deactivated at 95°C for 5 min and the pre-amplified-converted DNA was subjected to 10 × dilutions in ddH_2_O for MSP reaction.

### Statistical analysis

Correlations between methylation status and clinicopathological features were assessed using v2 or Fisher's exact probability tests as appropriate. All *P*-values presented are two-sided. A *P*-value <0.05 was regarded as statistically significant. Survival curves were generated using Kaplan–Meier method. All statistical tests were performed using MedCalc (version 9.2.0.1, MedCalc, Mariakerke, Belgium) and SPSS (version 16.0, SPSS Inc, Somers, NY, USA) software package.

## Results

### MicroRNAs selection following AZA treatment

To identify which miRNAs undergo specific epigenetic silencing at 5′ promoter flanking, we used a super-array RT–qPCR technique which can simultaneously detect miRNAs that have been silenced by epigenetic modification. This array consists of 46 miRNAs, which possessed a CpG island at the promoter region (identified by http://www.mirbase.org/ and http://www-bimas.cit.nih.gov/molbio/proscan/ – predicted sequence regions which contain a significant number and type of transcriptional elements that are usually associated with Pol II promoter sequences) and with two endogenous control small RNAs (RNU19 and RNU6B) for data normalisation. We treated HCT116 and HT29 CRC cell lines with AZA and found elevated expression of 12 miRNAs (Table 2). Following this, we determined which miRNAs would have potential role in the tumour microenvironment. On the basis of the literature, we found that miR-34b/c was downregulated in most of cancers including colorectal and have tumour suppressor properties (He *et al*, 2005; Chang *et al*, 2007; Hermeking, 2010). In addition, miR-148a was correlated to the tumour metastasis which involved a panel of cancer models (Lujambio *et al*, 2008). Thus, based on these previous findings, we chose two miRNAs (miR-34b/c and miR-148a) for further evaluation using MSP in CRC fresh frozen and faecal specimens. We avoided selection of certain miRNAs from our list that were upregulated following AZA treatment for two reasons; (i) some miRNAs have been reported previously to be overexpressed in CRC *vs* matched tissues and (ii) we particularly looked for screening markers which would detect cancer more accurately than the available screening markers and with a high degree of sensitivity.

### Expression of miR-34b/c and miR-148a following AZA treatment

**Figure 1:**
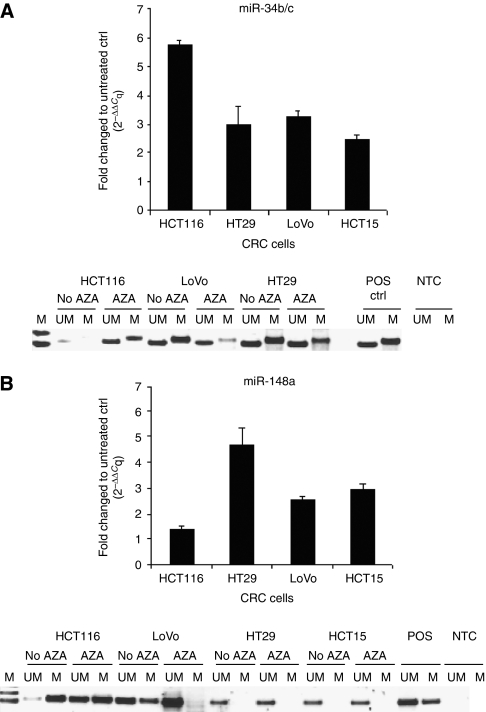
Methylation status of miR-34b/c and miR-148a in a panel of CRC cell lines assessed by RT–qPCR and methylation-specific PCR (MSP). Fold change expression of miR-34b/c (**A**, upper panel) and miR-148a (**B**, upper panel) following 10 *μ*M demethylating agent 5-aza-2′-deoxycytidine over 5 days of incubation in HCT116, HT29, LoVo and HCT15 assessed by RT-qPCR and by means of a 2^^−ΔΔ^*C*_q_ method. Corresponding methylation-specific PCR for miR-34b/c (**A**, lower panel) and miR-148a (**B**, lower panel) showing the decrease in methylation pattern following 10 *μ*M demethylating agent 5-aza-2′-deoxycytidine. Lane UM and M corresponded to unmethylated and methylated reaction respectively. Qiagen methylated and unmethlated control (ctrl) DNAs served as a reaction control for PCR. NTC=negative template control; POS=positive template control; AZA=5-aza-2′-deoxycytidine.

Having determined that miR-34b/c and miR-148a were involved in cancer progression, we analysed these miRNAs to ensure that what we observed with super array technique was paralleled with single RT–qPCR analysis. To assess the effects of miR-34b/c and miR-148a promoter methylation, four CRC cell lines (HCT116, HT29, LoVo and HCT15) were further treated with AZA for 5 days. We found that all CRC cell lines responded concordantly to the AZA treatment as shown in Figures 1A and B. For miR-34b/c, about a 6-fold change in expression level was observed followed by LoVo (3.3-fold), HT29 (3-fold) and HCT15 (2.5-fold; Figure 1A, upper panel). In contrast, in HT29 cells we found a strong increase in expression of miR-148a following AZA treatment (5-fold) followed by HCT15 (3-fold), LoVo (2.6-fold) and HCT116 cell lines (1.5-fold; Figure 1B, upper panel). These data suggested that the transcription of these miRNAs (miR-34b/c, miR-148a) were silenced by hypermethylation modification. The differences in expression levels of miRNAs following AZA treatment may also reflect the treatment efficiency. Next, we wanted to confirm whether the upregulated miR-34b/c and miR-148a expression observed by RT–qPCR was paralleled with MSP analysis. Restoration of miR-34b/c expression was more profound in LoVo and HT29 cells than HCT116 cells (Figure 1A, lower panel). In contrast, no methylation of miR-148a in HT29 and HCT15 cells was noticed, whereas more profound restoration of miR-148a expression was detected in LoVo than HCT116 cells as decreased in promoter methylation was detected by MSP (Figure 1B, lower panel).

### Analysis of promoter methylation of miR-34b/c in primary tumours and matched normal tissues using MSP

First, we assessed the promoter methylation of miR-34b/c by MSP in 81 neoplastic colonic mucosa and 42 matched normal tissues (Table 3a, upper panel).

**Figure 2:**
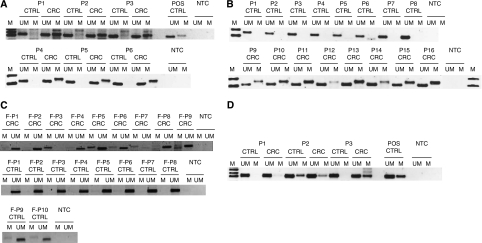
Methylation-specific PCR (MSP) reactions for the miR-34b/c and miR-148a promoter region in tumour and faecal specimens derived from CRC and/or normal individual. (**A** and **B**) MSP analysis for miR-34b/c in matched CRC samples. (**C**) MSP analysis for miR-34b/c in faeces of CRC (upper panel), colonoscopy negative individuals (middle panel) and high-grade dysplasia (lower pane). (**D**) MSP analysis for miR-148a in matched CRC tissues. Lane UM and M corresponded to unmethylated and methylated reaction, respectively. Qiagen methylated and unmethylated control (CTRL) DNAs served as a reaction control for polymerase chain reaction. NTC=negative template control; POS=positive template control; P=patients; F-P1=samples derived from faeces of CRC and normal individuals.

We found that 97.5% (*n*=79 out of 81) of neoplastic samples showed promoter methylation of miR-34b/c (Figures 2A and B). In contrast, promoter methylation of miR-34b/c was only detected in 14.3% (*n*=6/42; *P*<0.001; Figures 2A and B, Table 4) of matched normal colonic tissues. No correlation were found for miR-34b/c methylation pattern with clinicopathological status except for one significant correlation for pTNM stage, *P*=0.0110 (Table 4). However, this result is conflicting as only two samples were not positive for stage II cancer.

### Promoter methylation of miR-34b/c detection in faeces of CRC patient

Having confirmed that the expression of miR-34b/c is suppressed by methylation pattern in a large subset of CRC (97.5%), we wanted to confirm whether the same methylated pattern could be detected in faecal specimens. We used previously extracted faecal DNA for MSP for miR-34b/c methylation detection (Table 3b, lower panel; Kalimutho *et al*, 2010b) and used REPLI-g technology to enhance the detection rate of low abundant methylated alles in faeces. This assay would pre-amplify the low abundant methylated copies, which is the main drawback in faecal-DNA methylation analysis. We found that 75% (*n*=21 out of 28) of CRC cases were positive for promoter methylation of miR-34b/c (Figure 2C, upper panel, Table 5). At the same time, we analysed colonoscopy faecal DNA from negative patients and found that only 13% (*n*=5 out of 39) of cases had a positive methylation pattern for miR-34b/c. This result mirrored the data obtained for miR-34b/c in primary tissue samples (Table 4, Figure 2C, middle panel). Furthermore, we wanted to confirm whether the same methylated pattern could be observed in HGD. However, we found that only two cases were positive for miR-34b/c methylation, *n*=12 (Figure 2C, lower panel, Table 5).

### Promoter methylation of miR-148a and their correlation to clinicopathological features

Next, we analysed the promoter methylation pattern of miR-148a on the same tumour samples analysed for miR-34b/c. We also wanted to correlate miR-148a methylation pattern with clinicopathological features. No significant correlation was found between miR-148a methylation status with clinicopathological features except for a trend (although not significant) with female gender (*P*=0.08; Table 4), and with advanced age (*P*=0.101). We found that stage III (65%) and stage IV (75%) disease were more prone to have methylated pattern for miR-148a but this was not significant. Moreover, pT4 tumours were fully methylated (100%) compared with pT tumours in which only 65% were positive for methylation. Overall, 65% of CRC tumour tissues were positive for miR-148a methylation.

**Figure 3:**
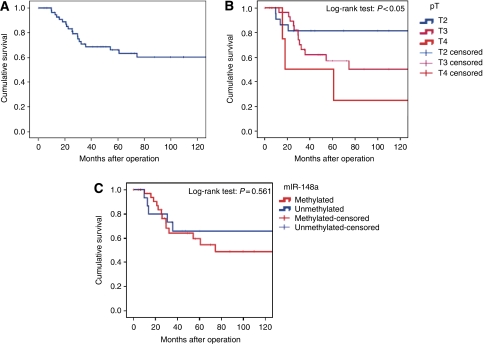
Box and Whisker plot and Kaplan–Meier disease-free survival analysis for CRC patients according to miR-148a hypermethylation pattern. (**A**) The 10-year survival rate (60%) of CRC patients was calculated using the Kaplan–Meier method. (**B**) Survival rate of CRC patients according to depth of invasion. The difference is statistically significant (log-rank test: *P*<0.05). (**C**) The prognostic value of miR-148a methylation status was with a trend towards lower survival rate in patients with methylated alleles (10-year survival probability: 48%) compared with unmethylated cases (10-year survival probability: 65%), log-rank test: *P*=0.561.

We also wanted to determine the prognostic value of miR-148a methylation status in this patient cohort. Surgical approach in patients with colon cancer consisted of standard hemicolectomy (via laparotomic or laparoscopic approach) with negative macroscopic resection margin and regional lymphadenectomy in all cases. The median number (range) of removed lymph nodes was 17 (3–56). In rectal cancer, laparotomic/laparoscopic low anterior resection or abdominoperineal resections with total mesorectal excision were performed; the median number of removed lymph nodes was 13 (range: 4–37). Overall survival was calculated according to the Kaplan–Meier method in 60 patients available for survival analysis, considering cancer related death as the end point. The median follow-up period was 46 months (range: 1–266 months). Comparison between survival curves was performed with the log-rank test. The 10-year survival rate of the entire series was 60% (Figure 3A). Survival was significantly related to the stage of the tumour, and depth of invasion was one of the most important prognostic factors (log-rank test: *P*<0.05; Figure 3B). Interestingly, we found a trend towards lower overall survival in the patients with methylated miR-148a alleles (10-year survival probability: 48%) compared with patients with unmethylated miR-148a alleles (10-year survival probability: 65% Figure 3C); however, the difference was not statistically significant (log-rank test: *P*=0.561) because of the small number of patients available for survival analysis.

## Discussion

Previous studies have shown that half of the miRNAs are located in or near CpG islands, which are transcriptionally regulated by DNA methylation process that varies between normal and tumour cells (Lujambio *et al*, 2007, 2008). For example, the methylation pattern of miR-124a is tumour specific, whereas miR-127 is methylated in both normal and tumour tissues (Saito *et al*, 2006; Lujambio *et al*, 2008), and let-7a-3 is methylated in normal tissues, whereas it shows a hypomethylation pattern in lung adenocarcinomas (Brueckener *et al*, 2007). However, the mechanism underlying the deregulation of miRNAs in cancer has not yet been fully elucidated.

With regard to this, we focused our study on the identification of miRNA with low expression in CRC and which may be involved in the development of CRC tumourigenesis. We evaluated expression levels of 46 miRNAs and found that 12 miRNAs were upregulated following AZA treatment. Of these, we decided to further investigate two miRNAs, miR-34b/c and miR-148a, which also previously been reported (Lujambio *et al*, 2008; Toyota *et al*, 2008); however, the clinicopathological features have not been fully evaluated in colorectal tumours. Two other main reasons for selecting these miRNAs are: (i) first, to evaluate the methylation pattern of miR-34b/c in tumours tissues in order to extend the work of Toyata *et al* (2008) and to further evaluate it in faecal samples. This would allow us to develop faecal-DNA-based markers for CRC screening; (ii) second, miR-148a was correlated to metastasis properties, yet no clinical correlation with methylation pattern is available for CRC. We also reasoned that miRNAs may be a better class of tumour marker because of their broad regulatory functions and the ability to measure their expression levels with far better accuracy than is currently achievable for mRNA.

Thus, to further elucidate the role of these miRNAs, four CRC cell lines were treated with AZA, which allows us to demonstrate that reduced expression of both miRNAs is directly related to promoter methylation. The most striking evidence from our study is that the CpG island of the promoter region of miR-34b/c are hypermethylated in 97.5% CRC cases, and this methylation is, therefore believed to be tumour specific for CRC. The high percentage of methylation pattern in these CRC cases, and the contribution of miR-34 family on the p53 network, suggest that miR-34b/c may be involved in the response to colorectal tumourigenesis. As induction of cell cycle arrest, senescence and apoptosis are the mechanisms of oncosuppression by miR-34 family (Chang *et al*, 2007; Hermeking, 2010), permanent inactivation due to epigenetic silencing may result in a selective advantage for cancer cell proliferation. The first report by Toyata *et al* (2008) on methylation pattern revealed that miR-34b/c was aberrantly hypermethylated in primary CRC tumours. They showed that epigenetically silenced miR-34b/c could possibly be involved in the early process of tumourigenesis. Besides, Corney *et al* (2010) recently reported downregulation of miR-34b/c in ovarian cancer. They found that miR-34b/c is significantly reduced in stage IV compared with stage III tumours. Accordingly, their data support our observation in which methylation patterns are inversely correlated towards tumour stage in CRC. Furthermore, the results obtained by this study combined with our own observation, could correlate miR-34b/c hypermethylation status into the development of screening markers for CRC detection.

We further evaluated miR-34b/c methylation in faecal specimens. Alteration in the genome that would lead to the progression of cancer could be detected using faecal specimens from CRC patients. Faecal DNA analysis was shown to represent a novel non-invasive method for CRC detection (Dong *et al*, 2001; Kalimutho *et al*, 2010b). We found that almost 75% of the CRC patients could be detected using faecal specimens. This is considered a good percentage to detect cancer using faeces as a screening marker, as methylated faecal DNA is almost inflexible to detect in the complex microenvironment of faeces. Furthermore, our data are based on a small group of faecal analysis, thus a large randomised blind study should take place to evaluate the actual sensitivity of this marker for CRC screening. In addition, the low percentage of CRC detection in faeces compared with 98% in tumour samples maybe because of the nature of the technique used for MSP observation. Hence, more advanced techniques, such as pyrosequencing or even quantitative-MSP using a specific probe of minor groove binder, would enhance the detection rate of miR-34b/c in faeces. However, as the methylation pattern of miR-34b/c shows almost no trend on tumour stage and possesses a high level of promoter methylation state in CRC, both in tumour and feaces, this would facilitate the feasibility of assaying faecal miR-34b/c as an additional test in clinical settings.

Next, we wanted to see whether the hypermethylation of miR-148a could correlate into clinicopathological features of CRC. The miR-148a expression is shown to be downregulated in human breast cancer and undifferentiated in gastric cancer (Lehmann *et al*, 2008; Katada *et al*, 2009). DNA methylation-associated silencing of miR-148 expression is identified in human cancer cell lines established from lymph node metastasis of colon, melanoma, and head and neck cancer, suggesting its role in the development of metastasis (Lujambio *et al*, 2008). Duursma *et al* (2008) have shown that overexpression of miR-148a leads to a reduction in the expression of *de novo* DNA-methyl-transferase-3b (DNMT3b) enzyme. The suppression of miR-148a, in contrast, induces increased expression of DNMT3b. The evidence shows that alteration of DNMT3b expression clearly contributes to CRC tumourigenesis. Lin *et al* (2006) as well Jin *et al* (2009) showed that the increased expression patterns of DNMT3b protein in Apc^Min/+^ increases colorectal carcinogenesis because of the hypermethylation of tumour suppressors genes. Also, Ahmed *et al* (2008) showed that reducing expression of DNMT3b in PC3 tumour cells induces the loss of methylation at the promoters of several tumour suppressor genes such as APC, RB1 and RER-*β*. Therefore, methylation at the promoter of miR-148a leads to increased expression of DNMT3b, which in turn inactivates its tumour suppressor function in most of the cancers. The correlation of our data with miR-148a promoter methylation pattern, together with Lujambio *et al* (2008), allows us to link the epigenetically silenced miR-148a with the progression to advanced tumour stage. For example, 65% (*n*=30 out of 46) of patients with stage T3, 100% (*n*=5) stage T4 and 78% (*n*=7 out of 9) with stage N2 of CRC, have a condition of promoter methylation of miR-148a. Very recently, Chen *et al* (2010) reported the downregulation of miR-148a and miR-152 in gastric and CRC by expression analysis in which they found a strong correlation between these two miRNAs. In support of our data, they also reported that no statistical correlation was found with clinicopathological features including sex, age, tumour location, histological grade, pN stage or lymphatic vessel invasion in gastrointestinal cancers. Despite this, we observed that the positive cases of promoter methylation of miR-148a have a lower survival rate than the negative cases (48 *vs* 65% at 10 years). In addition, the number of cases in our patient cohort is too small to investigate a potential impact of treatment stratified according to miR-148a methylation status, and this may represent a clinical limitation of this study. Further prospective studies may be designed in order to assess the potential clinical utility of multimodality treatments in tumours with more aggressive biology, identified by miR-148a expression.

In conclusion, as many CRC patients present with advanced disease, early detection leads to reduced mortality. Therefore, developing a miR-34b/c methylation assay as a diagnostic tool for early detection of CRC would have substantial clinical benefits. On the other hand, follow-up for the disease progression with targeted molecular markers such as miR-148a would enable classification of the prognosis of the disease as well as being a tool for therapy monitoring in patients having had CRC. Moreover, the reduced expression of miR-34b/c and miR-148a because of the epigenetic silencing could be considered as an important target of antineoplastic therapy development.

## Figures and Tables

**Table 1:** Primers list for methylation-specific PCR for miR34b/c and miR-148a, base pair and the annealing temperature (*T*_m_) of PCR

**Genes**	**Primer sets**	**PCR *T*_m_ (°C)**	**Base pair**
*miR 34b/c*, unmethylated	Forward: 5′-TGGTTTGTGGGGTTTTAAGG-3′	59	147
	Reverse: 5′-TCCCAACCCCAAACCCTA-3′		
*miR 34b/c*, methylated	Forward: 5′-ATTCGTTTCGTTTCGCGTTCGTTTC-3′	59	128
	Reverse: 5′-CTAAAACTAACTCTCTCGACCCCG-3′		
*miR 148a*, unmethylated	Forward: 5′-TTGGTAAAAGTTTAAATAATTATTGA-3′	54	115
	Reverse: 5′-CAACAAAAAAACTACAAAAATCACA-3′		
*miR 148a*, methylated	Forward: 5′-TTGGTAAAAGTTTAAATAATTATCGA-3′	56	117
	Reverse: 5′-CACAACAAAAAAACTACAAAAATCG-3′		

**Table 2:** miRNAs upregulation after the treatment with AZA

	**hsa-miR**	**HCT116**	**HT29**	**Expression in CRC**	**Functions**	**References**
1	15b	1.43	2.29	↑	Cell cycle	Xi *et al* (2006)
2	96	1.58	2.3	↑	DNA repairs	Bandrés *et al* (2006); Sarver *et al* (2009)
3	99a	3.85	2.23	↑	Cell inhibition	Chen *et al* (2008)
4	106a	1.23	2.15	↓↑	NA	Díaz *et al* (2008); Link *et al* (2010)
5	129	2.6	2.56	↓	Cell death	Bandres *et al* (2009); Huang *et al* (2009)
6	135b	1.75	2.02	↑	DNA repairs	Wang *et al* (2010)
7	146	1.09	2.63	↓↑	Anti metastatic	Hurst *et al* (2009); Slattery *et al* (2011)
8	148a	1.05	3.96	↓	Methylation	Lujambio *et al* (2008); Chen *et al* (2010)
9	181c	3.62	1.94	↓	Oncogenic suppresion	Hashimoto *et al* (2010)
10	219	1.31	3.41	↑	NA	Schetter *et al* (2008)
11	338	1.85	2.47	NA	NA	NA
12	34b/c	2.8	3.1	↓	Cell cycle and apoptosis	Toyota *et al* (2008)

Abbreviations: AZA=5-aza-2′-deoxycytidine; CRC=colorectal cancer; miRNAs=microRNAs; NA=data not available.

**Table 3:** Demographic of total patients analysed for both tumour and faecel samples

(A) Total of CRC tissue samples (*n*)	122
Median age both female and male (range)	68.0 (35–89)
	
*CRC samples*	
Male	48
Median age (range)	69.5 (42–84)
	
*CRC samples*	
Female	33
Median age (range)	62(35–89)
	
*Normal colon mucosa samples*	41
Male	28
Median age (range) years	68.5 (52–84)
	
(B) Total of faecal samples (*n*)	79
*Median age both female and male (range)*	62 (28–88)
Male	32
Median age (range)	62 (35–79)
	
*CRC patients*	28
Male	13
Median age (range) years	66 (49–79)
	
*High-grade dysplasia*	12
Male	8
Median age (range) years	62 (55–74)
	
*CRC/polyp-free subjects*	39
Male	11
Median age (range) years	58 (35–65)

Abbreviation: CRC=colorectal cancer.

**Table 4:** Association of miR-34b/c and miR-148a methylation pattern with clinocopathological features in tumour tissue specimen

	**Tumour tissue samples**					
	**miR-34b/c**			**miR-148a**		
	***n***	**Methylated**	**Unmethylated**	***n***	**Methylated**	**Unmethylated**
*Sex*	**81**			**78**		
Male	**48**	48	0	**46**	26	20
Female	**33**	31	2	**32**	25	7
	*P*=0.3181			*P*=0.0835		
						
*Tumour location*	**80**			**78**		
Proximal colon	**15**	15	0	**15**	9	6
Distal colon and rectum	**64**	64	2	**63**	42	21
	*P*=0.8112			*P*=0.8526		
						
*pT stage*	**79**			**78**		
T1	**2**	2	0	**1**	1	0
T2	**24**	24	2	**26**	15	11
T3	**48**	48	0	**46**	30	16
T4	**5**	5	0	**5**	5	0
	*P*=0.2272			*P*=0.2774		
						
*pTNM stage*	**81**			**78**		
II	**15**	13	2	**15**	9	6
III	**57**	57	0	**54**	35	19
IV	**9**	9	0	**9**	7	2
	*P*=0.0110^*^			*P*=0.6668		
Normal colon mucosa/control patients	**42**	6	36	**39**	10	29
	*P*<0.0001^*^			*P*=1.0000		

^*^P-value indicative significant data produced. The bold values indicate the total number of patients analyzed in this pilot study.

**Table 5:** Association of miR-34b/c methylation pattern with clinocopathological features in faecal specimen

	**Faecal samples**			
		**miR-34b-c**		
	***n*=79**	**Methylated**	**Unmethylated**	***P*-values**
*Sex*	28			
Male	13	10	3	
Female	15	11	4	*P*=0.8268
				
*Tumour location*	28			
Proximal colon	7	4	3	
Distal colon and rectum	21	17	4	*P*=0.497
				
*pTNM stage*	28			
0	5	3	2	
I	2	2	0	
II	6	3	3	
III	3	2	1	
Unknown	9	8	1	
Unclassified	1	1	0	
a	2	2	0	*P*=0.5055
				
High-grade dysplasia	12	2	10	
Normal colon mucosa/ control patients	39	5	34	*P*<0.0001^*^

a Histology report not available, as the patients underwent chemoradiotheraphy or underwent surgery at a different location.

^*^P-value indicative significant data produced.
